# Identification of JAK2 as a Mediator of FIP1L1-PDGFRA-Induced Eosinophil Growth and Function in CEL

**DOI:** 10.1371/journal.pone.0034912

**Published:** 2012-04-16

**Authors:** Bin Li, Guangsen Zhang, Cui Li, Dan He, Xinying Li, Chunfang Zhang, Faqing Tang, Xiyun Deng, Jingchen Lu, Youhong Tang, Ruijuan Li, Zhuchu Chen, Chaojun Duan

**Affiliations:** 1 Key Laboratory of Cancer Proteomics of Chinese Ministry of Health, Xiangya Hospital, Central South University, Changsha, People's Republic of China; 2 Division of Hematology, Institute of Molecular Hematology, the Second Xiang Ya Hospital, Central South University, Changsha City, Hunan, People's Republic of China; 3 Medical Research Center, Xiangya Hospital, Central South University, Changsha, People's Republic of China; 4 Division of Oncology, Xiangya Hospital, Central South University, Changsha, People's Republic of China; 5 Clinical Laboratory, Zhuhai Hospital, Jinan University, Zhuhai, People's Republic of China; 6 Department of Surgery, the University of Texas Health Science Center at Houston, Houston, Texas, United States of America; Emory University, United States of America

## Abstract

The Fip1-like1 (FIP1L1)-platelet-derived growth factor receptor alpha fusion gene (F/P) arising in the pluripotent hematopoietic stem cell (HSC),causes 14% to 60% of patients with hypereosinophilia syndrome (HES). These patients, classified as having F/P (+) chronic eosinophilic leukemia (CEL), present with clonal eosinophilia and display a more aggressive disease phenotype than patients with F/P (–) HES patients. The mechanisms underlying predominant eosinophil lineage targeting and the cytotoxicity of eosinophils in this leukemia remain unclear. Given that the Janus tyrosine kinase (JAK)/signal transducers and activators of transcription (Stat) signaling pathway is key to cytokine receptor-mediated eosinophil development and activated Stat3 and Stat5 regulate the expression of genes involved in F/P malignant transformation, we investigated whether and how JAK proteins were involved in the pathogenesis of F/P-induced CEL. F/P activation of JAK2, Stat3 and Stat5, were confirmed in all the 11 F/P (+) CEL patients examined. *In vitro* inhibition of JAK2 in EOL-1, primary F/P(+) CEL cells (PC) and T674I F/P Imatinib resistant cells(IR) by either JAK2-specific short interfering RNA (siRNA) or the tryphostin derivative AG490(AG490), significantly reduced cellular proliferation and induced cellular apoptosis. The F/P can enhance the IL-5-induced JAK2 activation, and further results indicated that JAK2 inhibition blocked IL-5-induced cellular migration and activation of the EOL-1 and PC cells in vitro. F/P-stimulation of the JAK2 suppressed cells led to a significantly reduction in Stat3 activation, but relatively normal induction of Stat5 activation. Interestingly, JAK2 inhibition also reduced PI3K, Akt and NF-κB activity in a dose-dependent manner, and suppressed expression levels of c-Myc and Survivin. These results strongly suggest that JAK2 is activated by F/P and is required for F/P stimulation of cellular proliferation and infiltration, possibly through induction of c-Myc and Survivin expression via activation of multiple signaling pathways, including NF-κB, Stat3, and PI3K/Akt.

## Introduction

An interstitial deletion on chromosome 4q12 results in the formation of the Fip1-like1 (FIP1L1)-platelet-derived growth factor receptor alpha fusion gene (F/P), which triggers the occurrence of chronic eosinophilic leukemia (CEL) [1]. F/P(+) CEL is characterized by hyperproliferation of clonal eosinophils and life-threatening organ damage, especially affecting the lungs and/or the heart, due to eosinophil degranulation of toxic mediators [2]. The F/P fusion protein acts as a constitutive activator of the transmembrane receptor protein-PDGFRA [3], [4], which activates several signal molecules such as PI3K, MEK, JNK, ERK1/2 and the Stats [5], [6], [7]. However, to date, it remains largely unknown which intracellular activated pathways and critical signal molecules underlie the F/P-mediated malignant phenotype of CEL.

Some studies on F/P(+) CEL have provided insights into the molecules that may contribute to this disease. A recent comparative proteomic analysis of eosinophils from F/P(+) patients, non-clonal hypereosinophilia syndrome (HES) patients and healthy donors indicated that SHP-1 tyrosine phosphatase activity was distinctively up-regulated in F/P(+) cells [8]. Another study investigating the effects of the pharmacological protein-tyrosine kinase inhibitor dasatinib found that the Lyn protein was excessively activated in F/P(+) CEL [9]. Since the pathogenesis of F/P(+) eosinophilia-associated atypical myeloproliferative neoplasms (Eos-MPN) is similar to that of BCR-Abl(+) chronic myeloid leukemia (CML), the involved signaling mechanisms may also be similar. Both diseases constitute a paradigmatic example of how constitutively active tyrosine kinases drive chronic leukemogenesis. JAK2 plays a vital role in the signal network mediating BCR-Abl(+) CML [10]. Recent results have indicated that JAK2, a downstream target of BCR-Abl, can maintain activated Lyn kinase in CML via the SHP-1 pathway, suggesting that JAK2 can mediate the BCR-Abl-induced activation of Lyn and SHP-1 kinase [11].

F/P induction of c-Myc promotes EOL-1 cellular proliferation, and the anti-apoptosis activity of F/P in eosinophils may be associated with high expression levels of cellular Survivin [7], [12]. Nonetheless, the mechanism by which F/P regulates c-Myc and Survivin is unknown. JAKs are cytoplasmic tyrosine kinases that participate in signaling initiated by a range of cell-surface receptors, including PDGFRA and a number of cytokine receptor superfamily members [13]. Eosinophil development during normal hematopoiesis occurs via the JAKs/Stats pathway [14], and c-Myc is a key target gene of JAKs during cytokine IL-5-induced eosinophil processes [15]. F/P has been shown in a mouse CEL model to cooperate with IL-5-dependent signaling to drive abnormal eosinophil infiltration and activation [16]. JAKs have also been shown to play a vital role in IL-5-dependent eosinophil migration and activation during the inflammatory reaction [17]. However, the role of JAKs in IL-5-induced chemotaxis and activation of EOL-1 cells has yet to be determined.

In this study, we initially examined whether JAK2 was involved in the F/P signaling pathway driving leukemia formation and whether it was stimulated by F/P synergistic with IL-5. Then, we investigated whether JAKs mediated the F/P-induced expression of c-Myc and Survivin. Finally, we investigated which JAKs-related signal transduction pathways, and particular downstream signal molecules, were aberrantly regulated in F/P(+) EOL-1 cells. The results indicate that JAK2 kinase is activated by F/P, and is required for F/P stimulation of cellular proliferation and infiltration by modulation of activities or expressions of multiple intracellular/nuclear molecules.

## Materials and Methods

The present study protocol was approved by the ethical committee at Xiangya Hospital of Central South University, Changsha, China.

### Patient Samples

A total of 28 patients, including 23 cases of HES, five of reactive eosinophilia (RE, two of which were eosinophilic gastroenteritis and three were ancylostomiasis) and five healthy volunteers, were included in this study. Karyotype analysis was normal. No abnormal chromatosomes, including those of PDGFRB, FGFR1 and JAK2, were detected in any of the cases [18]. The 23 HES patients met all the criteria for the diagnosis of HES, as proposed by Chusid [19]. Nested RT-PCR and fluorescence *in situ* hybridization (FISH) analyses were performed on all samples, and the F/P fusion gene was detected in the 11 HES/CEL patients, but not in the other 12 HES patients or other subjects. 10 of the 11 F/P(+) CEL cases had organ involvement (three of which had one affected organ and seven had at least two affected organs). Eosinophilic organ involvement/dysfunction comprised the spleen (n = 7), heart (n = 6), lung (n = 3), liver (n = 3), and the central nervous system (n = 1). The concentrations of serum IgE and IL-5 were normal in all 11 F/P(+) CEL patients. All these F/P(+) CEL patients had been treated with Imatinib, at initial daily doses ranging from 100 to 400 mg. All Imatinib-treated patients achieved complete haematological remission (CHR) (range:3–60 days), and ten of 11 patients with the F/P gene exhibited molecular remission (MR) within 1–19 months post-treatment (one patient was lost to follow-up). After obtaining informed consent, blood and bone marrow samples were collected from HES/CEL patients at the time of diagnosis at the Xiangya Hospital of Changsha.

### Cell Culture and Treatment

EOL-1 cells carried the WT F/P fusion oncogene (Braunschweig, Germany) [20]. Ba/F3 cells expressing T674I F/P resistant to Imatinib (IR) have been described previously [1]. After obtaining blood from the above-mentioned patients, polymorphonuclear leucocytes were separated by standard laboratory procedures [21]. Eosinophils were then separated by depletion of neutrophils with anti-CD16-coated magnetic microbeads using the magnetic cell separation system (MACS; Miltenyi Biotec GmbH, Bergisch- Gladbach, Germany) according to the following method(http://www.miltenyibiotec.com/download/datasheets_en/23/DS130_045_701.pdf). Eosinophils of greater than 95% purity were used in all functional experiments. All these cell lines and primary cells were maintained in RPMI-1640 medium supplemented with 10% fetal bovine serum (FBS) (Hyclone, USA) at 37°C in a humidified atmosphere of 5% CO_2_. The EOL-1 cells were treated with various concentrations of Imatinib mesylate (Gleevec, Novartis Switzerland) as indicated [1]. To determine the concentration of AG490 necessary to achieve maximal effects on JAK2 protein expression, a dilution series of AG490 in DMSO was prepared and applied to EOL-1 cells. An equal volume of DMSO was added to control wells. The results showed that 25 µM AG490 achieved ∼50% inhibition of JAK2 expression within 4 h, and 100 µM AG490 gave maximal inhibition (data not shown). According to these result, cells were selected to undergo JAK2 inhibition using different concentrations of AG490.

### Stimulation of Cells with IL-5

To determine whether synergism between the F/P and IL-5 to induces JAK2 kinase activation in human eosinophils. EOL-1 or primary F/P(+) CEL cells (PC) (5×10^5^/mL) were preincubated with or without Imatinib (10 nM) for 4 h and stimulated with IL-5 (5 ng/mL at 37°C for 0 to 5 min). The phosphylation level of JAK2 was detected by Western blot at the time points of 0, 2 and 5 min after IL-5 stimulation.

### Silencing of JAK2 Expression

EOL-1, PC and T674I F/P Imatinib-resistant (IR)cells were incubated with 1×10^−2^ µM of human JAK2 siRNA (Accell SMARTpool E-003146-00-0005, Thermo Fisher Scientific, Waltham, MA, USA) for 48 h, JAK2 mRNA and protein levels were measured using the RT-PCR and immunoblotting assays described above. A non-targeting scrambled siRNA (Accell Non-targeting pool D-001910-10-05, Thermo Fisher Scientific, Waltham, MA, USA) was used as a control in each sample.

### Cellular Proliferation and Apoptosis Assay

The cellular proliferation assay was performed using 3-(4, 5-dimethylthiazol-2-yl)-2, 5-diphenyltetrazolium bromide (MTT). Briefly, EOL-1, PC and IR cells were resuspended in RPMI-1640 medium (1.25×10^5^/mL) and dispensed into 96-well culture plates in 200 µl volumes. The cells were cultured and treated with either JAK2 inhibitor (AG490) or JAK2 siRNA as described above. At the end of the incubation, the cells were washed with PBS buffer and 50 µl of the MTT solution was added to each well. The plates were incubated for 4 h at 37°C and 5% CO_2_. The MTT solution was then removed and 150 µl of DMSO was added to each well. Finally, the absorbance (A value) was measured using a micro-culture plate reader at 490 nm. The cellular proliferation inhibition rate by AG490 or JAK2 siRNA was calculated according to the following equation: inhibition rate (%) = [(absorbance value of blank control group at 490 nm) – (absorbance value of the treated group at 490 nm)/absorbance value of blank control group at 490 nm]×100%. Apoptosis was determined by Annexin-V-FLUOS and propidium iodide(PI) double staining. The cell cycle was determined by propidium iodide nuclear staining according to the manufacturers' instructions (BD Biosciences Pharmingen, USA). Briefly, EOL-1, PC and IR cells were cultured and treated with either JAK2 inhibitor (AG490) at 0 µM, 25 µM, 75 µM, and 100 µM or JAK2 siRNA as described above. At the end of incubation, 1–10×10^5^ cells/mL were harvested, washed with PBS, and fixed with 70% ethanol. After incubation with propidium iodide for 30 min at 37°C, the cells were analyzed using a FACS Calibur flow cytometer (BD Biosciences). Data were collected and analyzed by the accompanying BD CellQuest software.

### Preparation of Nuclear Extracts

Cells were washed twice in 1 mL ice-cold PBS, resuspended in 400 µL hypotonic lysis buffer containing protease inhibitors, and incubated on ice for 20 min. Then, 12.5 µL of 10% NP-40 was added and the cell suspension was vigorously mixed for 15 seconds. The extracts were centrifuged for 2 min and the supernatants (cytoplasmic extracts) were discarded. Ice-cold nuclear extraction buffer (25 µL) was added to the pellets and incubated for 30 min with intermittent mixing. Extracts were centrifuged and the supernatant (nuclear extracts) transferred to pre-chilled tubes for storage at −70°C.

### Immunoprecipitation and Immunoblotting

Cells were rinsed twice with ice-cold PBS, solubilized in lysis buffer (50 mM Tris, pH 7.5, 1% NP-40, 150 mM NaCl, 2 mM EGTA, 1 mM Na3VO4, 100 mM NaF, 10 mM Na4P2O7, 1 mM phenylmethylsulfonyl fluoride, 10 µg/mL aprotinin, 10 µg/mL leupeptin), and centrifuged at 14000×g for 10 min at 4°C. The supernatant (cell extracts) was incubated on ice with anti-PDGFRA antibody (1∶1000 dilutions; Santa Cruz Biotechnology, USA) for 2 h. The immune complexes were collected following incubating with protein A-agarose (Roche, USA) at 4°C for 1 h. The beads were then washed three times with washing buffer (50 mM Tris, pH 7.5, 1% NP-40, 150 mM NaCl, 2 mM EGTA) and boiled for 5 min in SDS-PAGE sample buffer (50 mM Tris-HCl, pH 6.8, 2% SDS, 2% mercaptoethanol, 10% glycerol, 0.005% bromphenol blue). The solubilized proteins were separated by SDS-PAGE, transferred to a nitrocellulose membrane (Amersham Biosciences, Sweden), and detected by immunoblotting against phosphotyrosine (4G10) antibody. Whole-cell lysates were prepared from the cells, and western blotting was performed as described previously [22]. Blots were probed with the primary antibodies against phospho-JAK3(Tyr980)(p-JAK3), JAK3, phospho-Stat3(p-Stat3), Stat3, phospho-Stat5b(p-Stat5b), Stat5b, c-Myc, phospho-p85α(PI3K)/(Tyr467)(p-p85α), p85α, phospho-Akt1(Thr308/Ser473)(p-Akt1) and Akt1(1∶100–1∶2000 dilutions; Santa Cruz Biotechnology); phospho-Stat3(Tyr705)(p-Stat3), phospho-Stat5(Tyr694)(p-Stat5), phospho-JAK1 (pTyr 1022/1023)(p-JAK1), JAK1, phospho-JAK2 (Tyr1007/1008)(p-JAK2), JAK2, Survivin and β-actin (1∶1000–1∶2000 dilutions; Cell Signaling Technology, USA) followed by incubation with the secondary antibodies were used peroxidase- conjugated goat anti-mouse IgG or goat anti-rabbit IgG (1∶4000–1∶5000; Jackson ImmunoResearch Inc., USA) and enhanced chemiluminescent substrate. Nuclear extracts (500 ng protein per lane) were probed for phospho-p65 by Western blotting using antibodies to phospho-(NF-κB) p65/(Ser-536)(p-p65) (1∶100–1∶2000 dilutions; Cell Technology, USA) and PARP (control). Densitometry analysis was performed on exposed films using Quantity One v4.62 software (Bio-Rad, USA).

### Eosinophil migration and function Assay

The migration properties of EOL-1 and PC cells were analyzed in a 48-well microchamber (Neuroprobe, USA), in which the lower wells were filled with 28 µL of buffer alone or buffer containing 5 ng/mL IL-5. A fibronectin-coated polyvinylpyrrolidone-free filter (Neuroprobe) with 5 µm pores was placed over the lower wells and 50 µL of EOL-1 or PC cells at 4×10^6^ cells/mL was added to the upper wells. The chamber was incubated for 2 h at 37°C in a CO_2_ incubator. The polycarbonate filter was then removed and cells adhering to the upper surface were wiped off using a filter wiper. The filter was dried, fixed, and stained with Diff-Quick reagent (Baxter Diagnostics, USA). The cells in two randomly selected fields per well were counted using the Axiovert 25 microscope (Carl Zeiss, Germany). Each experiment included six replicate measurements. The chemotactic index (CI) was calculated as the number of cells that migrated in the experimental wells compared to those in control wells(which was set at 100%). EPO activities in EOL-1 and PC cells were measured using the following method. Briefly, 1×10^6^ cells/mL were plated on 6-well plates and incubated for 30 min in the presence of 5 ng/mL IL-5. EPO activity in EOL-1 or PC cells was measured by an enzyme-joint spectrophotometer, using the optical density (OD) value at an absorbance of 492 nm. The degranulation of EOL-1 or PC cells was detected using 180 µL of the 1×10^6^ cells/mL suspension in an Eppendorf tube. Following an initial incubation of 24 h at 37°C, IL-5 was added and the solution incubated for an additional 30 min, after which cells were collected by centrifugation (5000×g at 20°C for 5 min), washed with phosphate buffered saline (PBS), and resuspended in 200 µL Hank's salt solution. The cells were lysed by ultrasonication for 5 min, and the EPO activity was measured according Strath's method [23].

### Statistical Analysis

Data are presented as mean ±standard deviation (SD). Data were compared using the two-way analysis of variance(ANOVA) test or independent sample t test. P values less than 0.05 was considered statistically significant and were derived from 2-tailed statistical test. All statistical treatment was performed using SPSS 13.0 software.

## Results

### Excessive phosphorylation of JAK2, Stat3 and Stat5 in F/P(+) CEL patients

The 23 HES patients included 20 males and three females with a median age at diagnosis of 43 years (range: 15–73). The median white blood cell (WBC) count was 20.3×10^9^/L (range: 5–97) with an absolute eosinophil count (AEC) of 9.7×10^9^/L (range: 2.0–39). Serum IgE and IL-5 were within the normal range. The five RE patients had an AEC of 2.6×10^9^/L (range: 1.0–5.0), while the five healthy volunteers had an AEC of 0.2×10^9^/L (range: 0–0.5).

**Figure 1:**
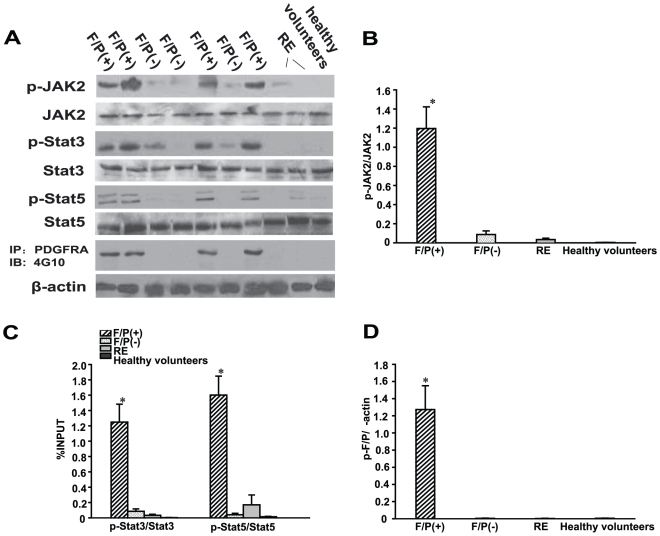
Activation of JAK2, Stat3 and Stat5 molecules in F/P (+) CEL patients. Cell extracts were prepared from blood samples of the cases with (F/P (+): F/P (+) CEL, F/P (−): F/P (−) HES, RE: reactive eosinophilia, and healthy volunteers) and subjected to western blotting or immunoprecipitation (IP). (**A**) Gel images of representative gel images. (**B–D**) Quantification of A. The results represent mean±SD of the cases(F/P (+) CEL [n = 11], F/P (−) HES [n = 12], RE [n = 5], and healthy volunteers [n = 5]). *P<0.05, compared to the other groups.

JAK2, Stat3 and Stat5 are closely related to the differentiation and proliferation of eosinophils. To determine whether these proteins were differentially activated in F/P(+) CEL patients, polymorphonuclear leucocytes and eosinophils were collected from all subjects and immunoblotted. Western blot results showed that phosphorylated JAK2 proteins were present at higher levels in F/P(+) CEL patients than in other eosinophilia patients lacking the F/P fusion gene or healthy volunteers (Figure 1A, B). The phosphorylated forms of Stat3 and Stat5 were also significantly higher in F/P(+) CEL patients, compared to the other groups (Figure 1A, C). However, total JAK2, Stat3 and Stat5 expression was not different among the groups. As expected immunoprecipitation of cell extracts with anti-PDGFRA antibody followed by immunoblotting with anti-phosphotyrosine (4G10), showed that phosphorylated F/P proteins were only detected in the 11 F/P(+) CEL patients (Figure 1A, D). Taken together these results indicate that F/P(+) CEL is uniquely characterized by excessive phosphorylation of JAK2, Stat3, and Stat5.

### Treatment of F/P(+) CEL patients and EOL-1 cells with Imatinib down-regulates phosphorylation of JAK2, Stat3 and Stat5 in a time- and dose-dependent manner

**Figure 2:**
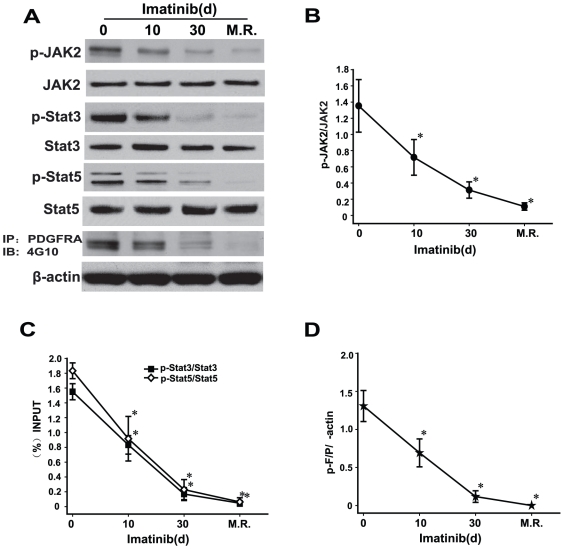
Inhibition of F/P tyrosine kinase by imatinib in CEL patients decreased phosphorylation of JAK2, Stat3 and Stat5 in a time-dependent manner. Blood samples were obtained from the F/P(+) CEL patients (n = 3) at pre-therapy (day 0), day 10, day 30 after therapy and at the time of molecular remission (M.R.). Eosinophils were separated by depletion of neutrophils with anti-CD16-coated magnetic microbeads using the magnetic cell separation system and whole-cell lysates were subjected to western blotting or immunoprecipitation (IP). (**A**) Gel images of representative gel images. (**B–D**) Quantification of A. Data (mean±SD) representative results derived from three independent experiments. *P<0.05, compared to day 0.

**Figure 3:**
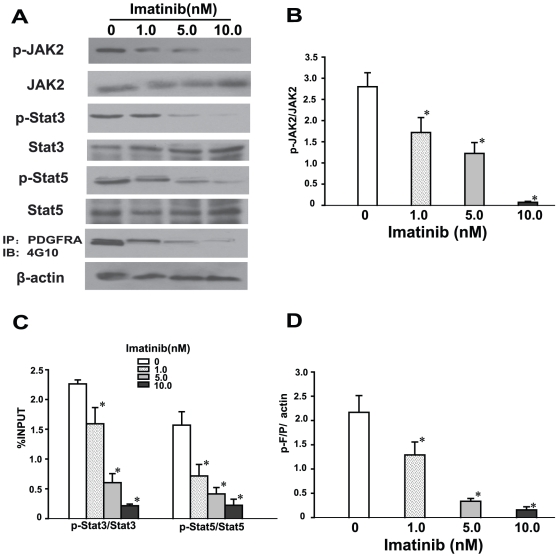
The specific F/P inhibitor imatinib down-regulated the phosphorylation level of JAK2, Stat3 and Stat5 in a dose-dependent manner. EOL-1 cells were treated with various concentrations of Imatinib. Whole-cell lysates were prepared and subjected to western blotting or immunoprecipitation. (**A**) Representative gel images. (**B–D**) Quantification of A. Data(mean±SD) representative results derived from three independent experiments. *P<0.05, compared to the untreated group.

The drug of choice for patients diagnosed with F/P(+) CEL is Imatinib, a specific inhibitor of F/P which often results in complete remission. All the 11 F/P(+) CEL patients in our study were also treated with Imatinib. Complete clinical remission was evidenced by abatement or disappearance of symptoms and/or changed laboratory values from the involved organ. To investigate whether phosphorylation of JAK2, Stat3, and Stat5 proteins were inhibited in F/P(+) CEL after treatment with Imatinib, peripheral blood samples were obtained at four different time-points: pre-therapy (day 0), post-therapy day 10 and day 30, and at the time of MR. In addition, we treated cultured EOL-1 cells with various concentrations of Imatinib. The results showed that the phosphorylation levels of JAK2, Stat3, and Stat5 were significantly decreased in both F/P(+) CEL patients and EOL-1 cells after treatment with Imatinib. The down-regulated phosphorylation levels of JAK2, Stat3, and Stat5 were correlated with the reduction in phosphorylation of the F/P in a time- and dose-dependent manner following Imatinib treatment (Figure 2, 3). These findings indicate that JAK2, Stat3, and Stat5 proteins lie downstream of the F/P signal.

### JAK2 inhibition blocks cellular proliferation in EOL-1, primary F/P(+) CEL cells (PC) and T674I F/P Imatinib-resistant cells(IR)

**Figure 4:**
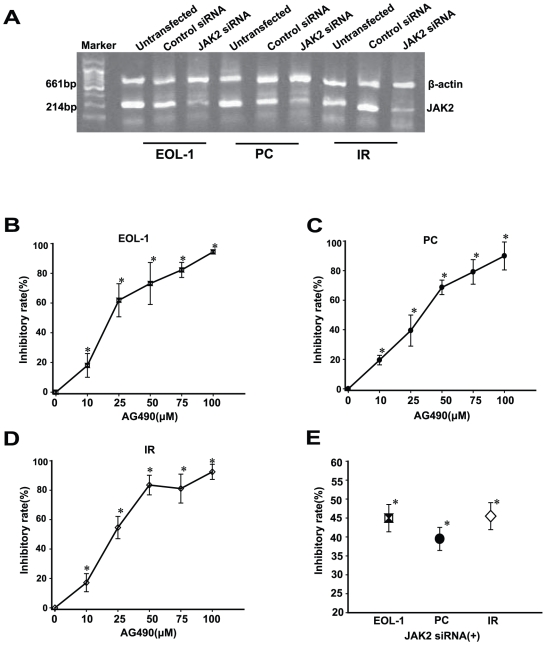
JAK2 inhibition or knock-down by AG490 or transfection of JAK2 siRNA significantly inhibited cellular proliferation in EOL-1, primary F/P(+) cells (PC) and T674I F/P Imatinib-resistant cells (IR). Cells were treated with various concentrations of AG490 or transfected with JAK2 siRNA. (**A**) Knock-down of JAK2 expression was confirmed by RT-PCR. (**B–E**) JAK2 inhibition or knock-down decreased cellular proliferation as assessed by MTT assay. Data(mean±SD) representative results derived from three independent experiments. *P<0.05, compared to untreated group or control siRNA.

The F/P oncoprotein is known to induce cellular proliferation and regulate prolonged survival of eosinophils. To explore whether the phosphorylation of JAK2 also contributes to cellular proliferation, we inhibited JAK2 activation with the specific inhibitor, AG490, or JAK2 siRNA and assessed the cellular growth using MTT assay (Figure 4). The results showed that the cellular proliferation inhibitory rate gradually increased with increasing AG490 concentration in EOL-1 cells. A similar result was also obtained with JAk2 knock-down (Figure 4B, E). We also observed that JAK2 inhibition or knock-down suppressed cellular proliferation in PC cells from patients (Figure 4C, E). More importantly, we found that cellular growth in IR cells was obviously repressed by JAK2 inhibition or knock-down (Figure 4D, E), indicating that a JAK2 inhibitor, to a certain extent, may represent an effective alternative therapy in Imatinib-resistant CEL.

### JAK2 inhibition induces cellular apoptosis of EOL-1, PC and IR cells

**Figure 5:**
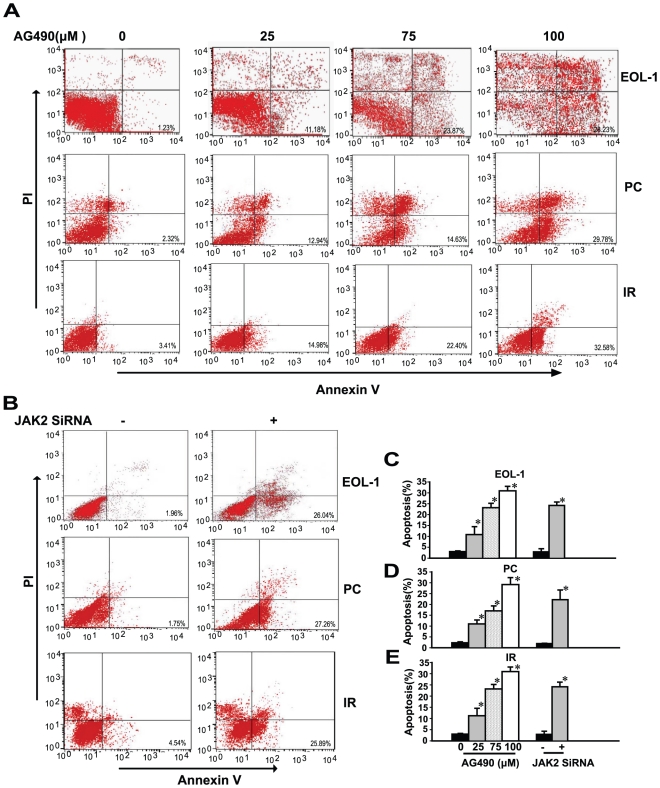
JAK2 inhibition or knock-down by AG490 or transfection of JAK2 siRNA induced cellular apoptosis in EOL-1, PC and IR cells. Cells were treated with various concentrations of AG490 or transfected with JAK2 siRNA. JAK2 inhibition increased cellular apoptosis assessed by flow cytometry using Annexin-V and PI. (**A–B**) Representative flow cytometry patterns. (**C–E**) Percentages of apoptotic cells treated with various concentrations of AG490 or transfected with JAK2 siRNA. Data(mean±SD) representative results derived from three independent experiments. *P<0.05, as compared to the untreated group or control siRNA.

The delay in apoptosis delay of eosinophils is another characteristic of F/P-mediated CEL. Therefore, we explored the role of JAK2 in delayed cellular apoptosis in F/P(+) CEL using the FACS assay. The results showed that EOL-1 cells underwent significant spontaneous apoptosis following exposure to the JAK2 kinase inhibitor, AG490, or transfection with JAK2 siRNA (Figure 5A, B, C). Similar results were also obtained in PC (Figure 5A, B, D) and IR cells (Figure 5A, B, E). These results indicated that the survival of F/P-mediated CEL cells was associated with activation of JAK2.

### F/P synergizes with IL-5 to induce JAK2 activation in EOL-1 and PC cells

**Figure 6:**
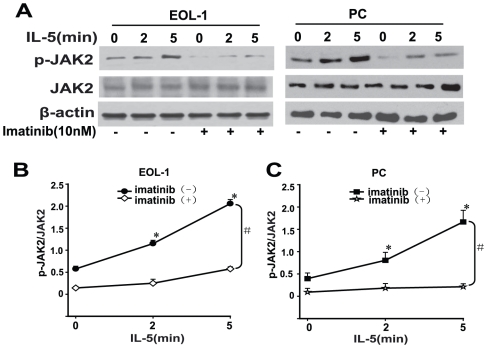
The synergistic role of F/P and IL-5 in inducing JAK2 activation in EOL-1 and PC cells. The EOL-1 or PC cells were preincubated with or without Imatinib for 4 h followed by treatment with 5 ng/mL IL-5 for 0 to 5 min. Whole-cell lysates were prepared and subjected to western blotting. (**A**) Representative gel images. (**B–C**) Quantification of A. Data(mean±SD) representative results derived from three independent experiments. * *P*<0.05, as compared to 0 minute. # *P*<0.05, compared to the differences between the Imatinib treatment and non-treatment for all the doses.

Our results suggest that JAK2 lies downstream of the F/P fusion protein. JAK2 is a known downstream effector of IL-5-stimulated signaling, which is implicated in the development, migration and activation of eosinophils. Therefore, we investigated whether the synergism between F/P and IL-5 to induced JAK2 activation using Western blotting. As expected, the results showed that IL-5 induced JAK2 activation in EOL-1 and PC cells, however, JAK2 activation was significantly inhibited by Imatinib, a specific inhibitor of the F/P (Figure 6), indicating a synergistic stimulation of JAK2 activation by F/P and IL-5 in these cells.

### JAK2 inhibition blocks IL-5-induced cellular migration and activation of EOL-1, PC and IR cells *in vitro*

**Figure 7:**
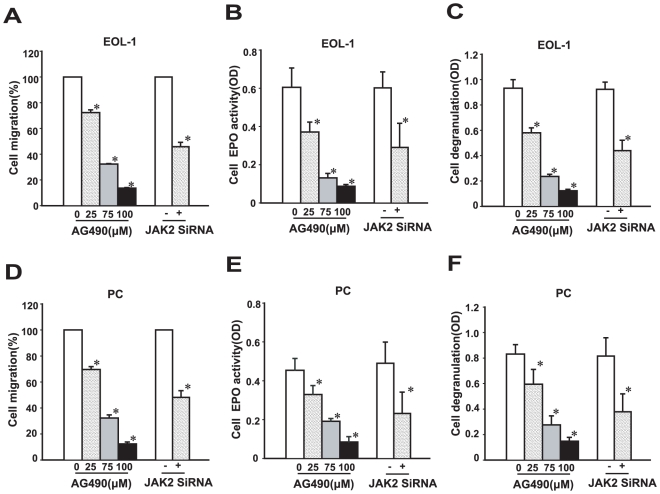
JAK2 inhibition blocked IL-5-induced the migration and activation of EOL-1 and PC cells. The EOL-1 or PC cells were treated with various concentrations of AG490 or transfected with JAK2 siRNA. (**A**) and (**D**) Percentage of migrated cell stimulated by IL-5. (**B**) and (**E**) EPO activity was measured by an enzyme-joint spectrophotometer, using the optical density (OD) value at an absorbance of 492 nm. (**C**) and (**F**) Cell degranulation was measured according Strath's method. Data(mean±SD) representative results derived from three independent experiments. *P<0.05, compared to untreated group or control siRNA.

Introduction of the F/P fusion gene to CD34+ hematopoietic stem cells(HSCs) induces myeloid proliferation and primes eosinophil differentiation, however, the development of eosinophil-associated end-organ infiltration and damage requires additional cytokines, especially robust expression of IL-5. Western blot results have showed that JAK2 was excessively activated by the F/P synergistic between and IL-5 (Figure 6). To explore the role of JAK2 in the migration and activation of EOL-1 and PC cells, IL-5 was applied as a chemoattractant and the effects of JAK2 inhibitor or knock-down were assessed. The results showed that JAK2 inhibition significantly blocked cells migration (Figure 7A, D) and depressed IL-5-induced cellular EPO activity (Figure 7B, E) and cell degranulation (Figure 7C, F) in a dose-dependent manner. These results indicate that activation of JAK2 enhances the invasive power of eosinophils, and maybe also be focus of F/P and IL-5 acting together in a synergistic manner to promote development of the CEL-like phenotype.

### Inhibition of JAK2 suppresses the phosphorylation of Stat3 and the PI3K/Akt signaling pathway in EOL-1 cells

**Figure 8:**
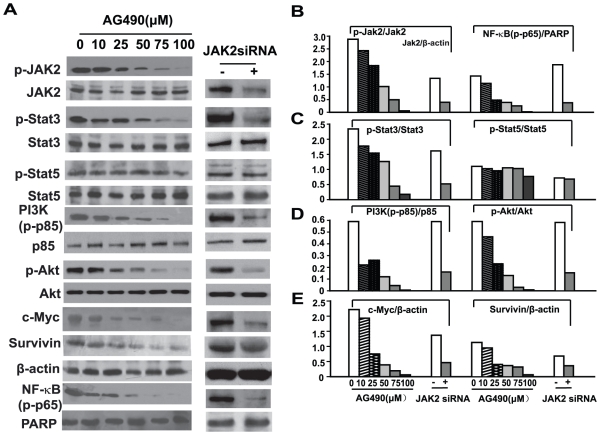
JAK2 inhibition resulted in the downregulation of NF-κB, c-Myc and Survivin expression which correlated with Stat3, PI3K/Akt deactivation. The EOL-1 cells were treated with various concentrations of AG490 or transfected with JAK2 siRNA. Whole-cell lysates were prepared and subjected to Western blotting or nuclear extracts were probed for phospho-p65 by Western blotting using antibodies to phospho-specific p65 and PARP. (**A**) **R**epresentative gel images. (**B–E**) Quantification of A.

The above data demonstrate that JAK2 kinase was essential for F/P-induced CEL cellular proliferation, survival and activation. We next investigated which signal transduction pathways involving JAK2 were disrupted in F/P(+) EOL-1 cells. These cells were treated with different concentrations of AG490 and evaluated by western blot with antibodies to the various molecules related to JAK2 (Figure 8A, B). Phosphorylation of Stat3 was found to decrease gradually with increased AG490 concentration. The phosphorylation status of Stat5 showed no obvious changes at low AG490 concentrations, but showed a slight decrease in the phosphorylated form at high concentration (100 µmol/L) (Figure 8A, C). JAK2 inhibition by AG490 also caused a dramatic and dose-dependent decrease in the phosphorylation level of PI3K and Akt (Figure 8A, D). To confirm these findings, we examined the effects of JAK2 knock-down by JAK2 siRNA in EOL-1 cells. Phosphorylation of Stat3, PI3K and Akt were considerably reduced in JAK2 knock-down cells, as compared with non-silenced cells (Figure 8A, C, D). In contrast, JAK2 knock-down had no obvious effect on Stat5 phosphorylation (Figure 8A, C). These results indicate that JAK2 can mediate the F/P-induced activation of Stat3 and the PI3K/Akt pathway, but is not the principal mediator of F/P-induced Stat5 activation.

### Inhibition of JAK2 downregulates the expression of multiple target genes including NF-κB, c-Myc and Survivin in EOL-1 cells

NF-κB is believed to play a role in the migration and activation of eosinophils. To examine the effect of JAK2 on NF-κB activity and further assess the role of JAK2 in the F/P-induced expression of c-Myc and Survivin, EOL-1 cells were treated with various concentrations of the JAK2 inhibitor AG490 and immunoblotted. The nuclear fractions were assessed for the phosphorylation level of the NF-κB p65 subunit and the whole protein extracts were assessed for c-Myc or Survivin. The results showed that p65 phosphorylation in the nuclear fraction, and c-Myc and Survivin expression in the whole cell were dramatically decreased by JAK2 inhibition in a dose-dependent manner. JAK2-siRNA transfected EOL-1 cells also showed significant reduction in the expression of the above genes, as compared with the non-silenced controls (Figure 8A, B, E). These results indicate that c-Myc and Survivin are both downstream targets of JAK2, and that JAK2 has an important role in maintaining NF-κB sustained activity in F/P(+) eosinophils.

## Discussion

The F/P fusion protein, acting as a constitutively active tyrosine kinase, triggers a series of intracellular molecular events leading to the occurrence of CEL. The mechanisms underlying the predominant eosinophil lineage targeting and eosinophil cytotoxicity in this leukemia remain unclear. In this study, we have shown for the first time that JAK2 is involved in the F/P-stimulation of cellular proliferation and infiltration via multiple signaling pathways. Several lines of evidence support this conclusion. First, by evaluating the effects of the specific inhibitor Imatinib *in vivo and in vitro*, we demonstrated that JAK2, as well as Stat3 and Stat5, are downstreams of the F/P fusion gene. Second, JAK2 inhibition by AG490 or siRNA dramatically inhibited cellular proliferation and induced cellular apoptosis of the EOL-1, primary F/P(+) CEL (PC) and T674I F/P Imatinib-resistant CEL (IR) cells. Third, JAK2 kinase was the synergized downstream of the F/P and IL-5, and JAK2 inhibition significantly blocked IL-5-induced cellular migration and activation of EOL-1 and PC cells. Fourth, specific inhibition of JAK2 significantly suppressed the phosphorylation of Stat3, but had no obvious effect on the phosphorylation level of Stat5. Finally, JAK2 inhibition led to a dose-dependent decreases in PI3K, Akt and NF-κB activity and reduced F/P-induced expressions of c-Myc and Survivin.

JAK proteins are central components of hematopoietic cell production and biological function, and effective targets of myeloproliferative neoplasms [24], [25]. A recent study showed that JAKs induction of c-Myc is critical to IL-5 stimulation of eosinophil cell proliferation and inhibition of apoptosis [15]. Our study showed that all 11 CEL patients carrying the F/P gene exhibited more intense phosphorylation of JAK2 than the other eosinophilia cases without this fusion gene. There were no statistical differences in the expressions of phospho-JAK1 or phospho-JAK3 (data not shown). Phosphorylation of JAK2 was inhibited by Imatinib in a time- and dose-dependent manner. Collectively, these findings suggest that JAK2, and not JAK1 or JAK3, participates in the pathogenesis of F/P(+) CEL. Intriguingly, eosinophilic gastroenteritis patients express high levels of phospho-JAK3, which is coincident with the finding that JAK3 activation is critical for airway eosinophilic inflammation, as in asthma and rhinitis [26]. In addition, the F/P-induced activation of Stat3 and Stat5 observed in our study was consistent with previous findings [1], [5], [27], [28].

EOL-1 cells harbor the F/P fusion gene, which inhibits eosinophilic precursor cells from differentiating into mature eosinophils, but also causes transformation into leukemia cells [20]. F/P-transformed cells have been demonstrated to undergo cytokine-independent proliferation. One of the major mechanisms of F/P(+) CEL malignancy is the up-regulation of c-Myc induced by F/P [7]. The F/P oncoprotein has also been implicated in the prolonged survival of eosinophils in CEL [7], [29], which may result from the abnormally high expressions of c-IAP and Survivin [12]. However, the molecular process by which the F/P signal elicits rapid changes in gene expression in eosinophils is not well understood. Multiple signal molecules, including Stats, PI3K, and ERK1/2 proteins, have been shown to be important, but not sufficient for mediating the F/P oncogenic transformation function [5]. In the present study, JAK2 inhibition significantly reversed F/P-induced colony formation and promoted EOL-1 cellular apoptosis. These events were accompanied by dose-dependent decreases in c-Myc and Survivin expression level. Thus, JAK2 acts as another crucial intracellular signal protein in F/P-mediated CEL.

Stats are latent cytoplasmic transcription factors that are generally considered to be JAKs-dependent, especially in hematopoiesis and some hematopoietic diseases. Stat5 was the first Stat protein to be associated with activation by F/P in CEL, and subsequent evidence has shown that it is necessary for F/P-induced colony formation [1], 5. The second Stat molecule to be identified as a target of F/P was Stat3, and its activation has been implicated in signal propagation of the F/P protein [27], [28]. However, the molecular mechanism by which F/P activates Stat5 and Stat3 remains unclear. The results from our study showed that JAK2 is involved in the F/P-induced activation of both Stat5 and Stat3. Phosphorylation of Stat5 was slightly affected(inhibited) by high concentration of the JAK2 inhibitor, AG490, or JAK2 knock-down by siRNA. These findings suggest that activation of Stat5 by F/P may occur to some extent through JAK2, but primarily occurs via another unidentified kinase. Considerable evidence exists to suggest that some activation of Stat5 occurs independently of the JAK2 [30], [31]. Our results also showed that the phosphorylation of Stat3 was decreased in a dose-dependent manner by JAK2 inhibition. Stat3 has been characterized as a central molecule of JAK2 intracellular signaling in solid tumor oncogenesis [32]. The development of eosinophil-associated end-organ infiltration and damage with release of cytoplasmic toxic mediators are the key features in CEL patients carrying the F/P gene, and are associated with poor prognosis due to multiple-organ failure [2], [33]. Mouse models of F/P or IL-5 overexpression revealed that neither molecule alone is sufficient to induce substantial tissue eosinophil infiltration or end-organ impairment, but together result in a severe, rapidly progressive disease resembling CEL [16]. Furthermore, the severity of F/P(+) CEL in humans has been associated with polymorphic variation at the IL-5 receptor A locus [34]. In this study, we found that JAK2 was excessively activated by the F/P in synergism with IL-5 in EOL-1 and PC cells. Thus, we used IL-5 as a chemoattractant to investigate whether JAK2 is involved in the chemotaxis of EOL-1 and PC cells *in vitro*. The results indicated that JAK2 activation is an important mediator of cell movement and activation stimulated by IL-5 *in vitro*. Although the molecular profile of JAK2 interactions generating signal leading to cell infiltration and activation remains obscure, our study showed for the first time that JAK2 maybe an alternative and feasible target for inhibiting F/P(+) eosinophil-associated tissue infiltration and dysfunction. The coexistence of T-cell clonality and the F/P fusion gene in 5%–28% of CEL patients may provide insight into the complex pathogenesis, but also indicates that IL-5 may be the most relevant cytokine in the pathogenesis of F/P-mediated CEL [35], [36]. It is rational to consider that JAK2 may be the vital downstream kinase activated by F/P converged with IL-5-stimulated intracellular signals in CEL cells [37], and that excessive phosphorylation of JAK2 may promote higher levels of eosinophil infiltration and activation in CEL by activating signal cascades that are different from those in normal eosinophil biological function.

NF-κB activation has been demonstrated to up-regulate the ICAM expression of EOL-1 cells, mediating cellular migration and adhesion [38]. In addition, NF-κB regulates the expression of key proinflammatory cytokines and other genes in activated eosinophils. Recent reports have shown that NF-κB is required in EOL-1 cells for increased expression and constitutive activation of protein kinase C-delta, which induces cell recruitment and migration [39], [40]. Therefore, the effect of JAK2 on NF-κB activity was observed in EOL-1 cells in our study, and western blot results showed that NF-κB activity was decreased in a dose-dependent manner when JAK2 was inhibited. These results indicate that NF-κB is another F/P-related signal molecule that lies downstream of JAK2. Moreover, NF-κB may be one of the principal mediators of eosinophil cellular infiltration and end-organ impairment which occur in F/P(+) CEL patients.

Although activation of Stat5 was able to induce cytokine-independent proliferation of EOL-1 cells, the colony size of transduced cells produced by constitutively active Stat5 was dramatically smaller than those produced by F/P-expressing cells. These results indicated that additional signaling molecules are a requisite for the clinical phenotype of F/P(+) CEL. Indeed, both PI3K and ERK1/2 are excessively activated during F/P-mediated eosinophil progenitor expansion and abnormal differentiation [5]. Our findings that JAK2 inhibition abated PI3K and Akt activity suggests that JAK2 can evoke pronounced activation of the PI3K/Akt signal pathway under conditions of F/P-stimulation. Recent studies have similarly indicated that activation of the JAK2/PI3K/Akt signaling pathway can effectively promote cellular proliferation, thereby contributing to the pathogenesis of hematopoietic malignancies [30], [41]. Moreover, the PI3K/Akt signaling pathway participates in eosinophil migration and degranulation stimulated by different chemotaxins [42], [43]. It has been suggested that distinct mechanisms underlie EOL-1 and normal eosinophil activation [8], [40]. In support of this, our results indicated that the aberrant activation of EOL-1 cells may be due to activation of the PI3K/Akt signaling pathway and NF-κB induced by the F/P via JAK2 kinase, but is not solely dependent on the JAKs/Stats signaling pathway.

**Figure 9:**
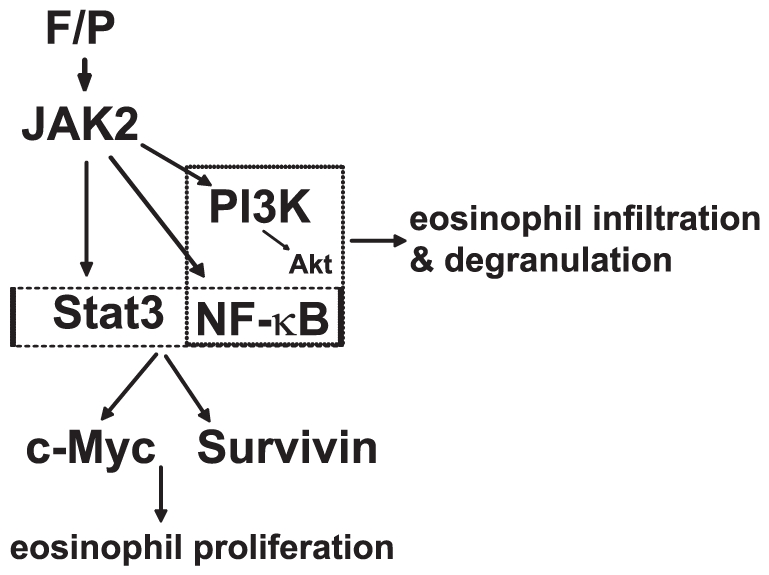
A model for the role and possible mechanism of JAK2 in F/P-induced CEL. JAK2-mediated activation of the PI3K/Akt signaling cascade and NF-κB kinase facilitates eosinophil activation and expression of target genes of JAK2 (c-Myc and Survivin) via the Stat3 and NF-κB signaling pathways, ultimately promotes cell proliferation.

In this study, our findings demonstrate that in the EOL-1 cell, JAK2 is able to control both the activities and gene expression of several different signaling molecules, including Stat3, PI3K, Akt, NF-κB, c-Myc and Survivin. This molecular profile is distinctive between the development and activation of EOL-1 cells and that of normal eosinophils induced by specific cytokines via the JAKs pathway (Figure 9). The transcription factors, NF-κB and Stat3, were previously characterized as critical to various aspects of the tumorigenic process in a number of malignancies, and shown to be functioning individually or synergistically. c-Myc is prominent amongst the target genes of both Stat3 and NF-κB. In contrast, the anti-apoptosis Survivin gene is promoted by Stat3, but not NF-κB [44], which is in accordance with the slight contribution of NF-κB to delayed apoptosis of EOL-1 cells [40].

Our findings reveal that JAK2 is a key target of the F/P fusion protein and underscores the importance of JAK2 signaling in the F/P-induced cellular proliferation, survival and infiltration events that manifest as CEL. JAK2 mediates the F/P-induced expression of c-Myc and Survivin, possibly through activation of multiple signaling pathways, particularly Stat3, PI3K/Akt and NF-κB. The F/P-induced phosphorylation of Stat5 appears to principally occur through another unknown signalling pathway, as opposed to JAK2 which regulates F/P-induced Stat3. Collectively, this evidences indicates that the pathogenesis of F/P(+) CEL is correlated with aberrantly regulated intracellular signaling pathways. Inhibition of the F/P-induced signal proteins might represent an effective alternative therapeutic approach. As such, JAK2 inhibition will be an excellent strategy to manage F/P(+) CEL patients who have become resistant or intolerant to Imatinib/dasatinib and other potent tyrosine kinase inhibitors. Moreover, as it is reported that dual inhibition of JAK2 and Stat5 enhances killing of myeloproliferative neoplasia cells [45], JAK2 inhibitors are likely to produce more benefit when combined with Stat5 inhibitors in the treatment of F/P(+) CEL. Future studies on the “cross-talk” between the signal molecules involved in F/P(+) CEL will facilitate a deeper understanding of the pathophysiology of this uniquely malignant HES/CEL caused by F/P.
